# Prolonged Mechanical Ventilation and Extubation Failure in Children and Adolescents Undergoing Cardiac Surgery

**DOI:** 10.21470/1678-9741-2023-0281

**Published:** 2025-02-05

**Authors:** Alessandra Muniz Pereira da Costa, Luziene Alencar Bonates dos Santos, Edinely Michely de Alencar Nelo, Lívia Barboza de Andrade

**Affiliations:** 1 Instituto de Medicina Integral Professor Fernando Figueira, Recife, Pernambuco, Brazil

**Keywords:** Mechanical Ventilation, Pediatric Cardiac Surgery, Extubation, Children

## Abstract

**Introduction:**

Mechanical ventilation (MV) is one of the factors that may be associated with postoperative complications of cardiac surgeries. This study aimed to verify the clinical and biological factors related to prolonged MV and extubation failure in children and adolescents submitted to cardiac surgeries.

**Method:**

This retrospective cohort included all patients aged between 0 and 15 years at the Unidade de Recuperação Cardio-Torácica Pediátrica who were submitted to the first extubation after cardiac surgery. Those tracheostomized and under MV before the surgery or who suffered accidental extubation were excluded. The following data was collected — age, weight, and sex; body mass index (BMI); heart disease; surgical severity (Risk Adjustment for Congenital Heart Surgery-1); hospitalization period and length of stay at intensive care unit; MV, cardiopulmonary bypass, and anoxia duration; use of continuous sedation (midazolam and/or fentanyl); pulmonary hypertension; nitric oxide use; Down syndrome, extubation site, and failure. The outcomes were prolonged MV and extubation failure.

**Results:**

A total of 233 patients were included — 79 (33.9%) aged below 12 months, 47 (20.2%) had Down syndrome, and 215 (92.3%) presented low BMI. Down syndrome patients and those under continuous sedation in the immediate postoperative period presented a higher risk of prolonged MV (*P*<0.001). Moreover, patients aged below 12 months (*P*=0.048) and those under prolonged MV (*P*=0.006) presented the highest risk of extubation failure.

**Conclusion:**

Patients with continuous sedation or Down syndrome required longer MV. In addition, children younger than 12 months or under prolonged MV presented a high extubation failure rate.

## INTRODUCTION

In Brazil, of the 29,000 children with congenital heart disease (CHD) born annually, about 6% (1,740) die in the first year of life^[1,2]^. These patients present morbidity rates that are influenced by several mortality risk factors. Most are related to surgery and the postoperative period, such as the prolonged mechanical ventilation (MV) use that may lead to ventilator-associated pneumonia^[3]^.

The literature is unclear in defining prolonged MV during the postoperative period of pediatric cardiac surgery. Most studies consider early extubation (or fast-tracking) when patients are extubated within six hours after the procedure, and prolonged MV when the extubation occurs after 24 hours^[4,5,6]^. Also, some studies recommend extubating patients in the operating room (OR) or within 24 hours to avoid prolonged MV, a risk factor for extubation failure. However, it is unclear which patients would need prolonged MV or which risk factors would be associated with extubation failure^[4]^.

Most pediatric cardiac surgery centers recommend immediate extubation in the OR^[4,7]^, Gaies et al.^[4]^ demonstrated in their study that the strategy of early extubation can be performed in the majority of patients, which can lead to a lower rate of extubation failure. However, its recommendation and the criteria to determine which patients could not undergo early extubation are unclear. Also, Cove et al.^[8]^ observed that late extubation after cardiac surgery hinders rehabilitation and increases hospital costs, whereas early extubation decreases the hospitalization period and total cost^[9]^. Conflicting data on risk factors for prolonged MV demonstrates the difficulty in reaching a consensus, mainly due to systemic and cardiopulmonary factors of patients undergoing cardiac surgeries^[10]^. Thus, this study aimed to associate clinical and biological factors with prolonged MV and extubation failure in children and adolescents submitted to surgical correction for CHD.

## METHODS

### Study Design

This observational retrospective cohort study^[11]^ was conducted between March 2020 and December 2022 at the Pronto-Socorro Cardiológico Universitário de Pernambuco Professor Luiz Tavares (PROCAPE), Universidade de Pernambuco (Pernambuco, Brazil). The study was approved by the research ethics committee of the Instituto de Medicina Integral Prof. Fernando Figueira (no. 49901821.5.3001.5192).

### Study Sample

The study included all children and adolescents aged between 0 and 15 years admitted to the Unidade de Recuperação Cardio-Torácica Pediátrica at PROCAPE, from January 2017 to December 2021, who underwent palliative or corrective cardiac surgery and with the first extubation. Those tracheostomized (patients who already had an artificial airway prior to the procedure), who suffered accidental extubation, or were under MV before cardiac surgery were excluded. From March 2020, patients were only submitted to the surgery if they tested negative for Coronavirus disease 2019 (reverse transcription polymerase chain reaction test) and did not present any symptoms related to acute respiratory disorder or influenza.

### Procedures

The following variables were collected: anthropometric and biological data (sex, age, and weight, body mass index, and nutritional status according to the World Health Organization classification [low, appropriate, or overweight according to age]); hospitalization period and length of stay at the intensive care unit (ICU); heart disease (cyanotic, acyanotic, left obstructive, or others); MV, cardiopulmonary bypass (CPB), and anoxia duration; need for continuous sedation (midazolam or fentanyl [or both]) in the immediate postoperative period; spontaneous breathing trial; Risk Adjustment for Congenital Heart Surgery (RACHS-1) score^[12]^; pulmonary hypertension (PH) (≥ 20 mmHg); nitric oxide use; Down syndrome (DS); and variables associated with the post-extubation period (*e.g.*, extubation site and failure). The variables were collected from electronic medical records and OR logbooks. Data were plotted into a spreadsheet (double-checked by different researchers at different times) and compared to correct any errors.

### Statistical Analysis

Bi and multivariate analyses encompassed Pearson's chi-square test, Fisher's exact test, and the Royston test of trend proportions to identify factors associated with prolonged MV (> 24 hours) and extubation failure. Statistical significance was set at *P*<0.05.

## RESULTS

We collected data from 265 patients; 32 were excluded: five were tracheostomized, 17 were under MV before surgery, and 10 were accidentally extubated. In addition, of the 233 patients, 138 (59.2%) had prolonged MV, and 22 (9.4%) had extubation failure in < 48 hours. Baseline characteristics are presented in Table 1.

**Table 1.** Baseline characteristics (n = 233).

Variables		N (%)
**Down syndrome**		47 (20.2)
**Sex**		
Female		121 (51.9)
Male		112 (48.1)
**BMI category (according to age)**		
Low		215 (92.3)
Appropriate		17 (7.3)
Overweight		1 (0.4)
**RACHS-1 categories**		
1		58 (24.9)
2		104 (44.6)
3		68 (29.2)
4		3 (1.3)
**Cardiopulmonary bypass**		161 (69.1)
**Spontaneous breathing trial**		110 (47.2)
**Extubation failure**		22 (9.4)
**Anoxia**		159 (68.2)
**CHD diagnosis**		
Acyanotic		144 (61.8)
Cyanotic		57 (24.5)
Left obstructive		20 (8.6)
Other		12 (5.2)
**Extubation site**		
Operating room		31 (13.3)
URCTPED		202 (86.7)
**Age (months)**		
< 12		79 (33.9)
12 to 24		53 (22.7)
> 24		101 (43.3)
**Mortality**		4 (1.7)
**Numerical variable**	**N**	Median (P25 to P75*)
CPB (min)	161	65.0 (50.0 to 05.0)
Total hospital stay (days)	233	13.0 (7.0 to 20.0)
Anoxia (min)	159	47.0 (34.0 to 70.0)
Age (months)	233	19.0 (8.0 to 51.0)

* P25 to P75=percentile 25 to percentile 75

BMI=body mass index; CHD=congenital heart diseases; CPB=cardiopulmonary bypass; RACHS-1=Risk Adjustment for Congenital Heart Surgery; URCTPED=Unidade de Recuperação Cardio-Torácica Pediátrica

Forty-seven (20.2%) subjects were DS patients with an average age of one year and seven months, of whom 27 (57.4%) had some degree of PH.

The highest frequency of RACHS-1 was in categories 2 and 3, and 161 patients underwent CPB (Table 1). The number of patients requiring MV for > 24 hours, failing the first extubation attempt, and who underwent spontaneous breathing trials before extubation are shown in Table 1. The ICU stay ranged from one to 116 days (Table 1).

The possible risk factors for prolonged MV were high RACHS-1 categories (*P*<0.001), DS patients (*P*<0.001), nitric oxide use (*P*=0.081), PH (*P*=0.016), continuous sedation in the immediate postoperative period (*P*<0.001), and age < 12 months (*P*<0.001) (Table 2).

**Table 2.** Bivariate analysis of the association between prolonged mechanical ventilation and different variables (n = 233).

	Prolonged MV		P-value*
	Yes	No	
**Variable**	**N (%)**	**N (%)**	
**Neonate**			0.098
Yes	7 (87.5)	1 (12.5)	
No	131 (58.2)	94 (41.8)	
**CHD diagnosis**			0.695
Acyanotic	81 (56.3)	63 (43.8)	
Cyanotic	36 (63.2)	21 (36.8)	
Left obstructive	13 (65.0)	7 (35.0)	
Other	8 (66.7)	4 (33.3)	
**BMI category (according to age)**			0.627
Low	126 (58.6)	89 (41.4)	
Appropriate	11 (64.7)	6 (35.3)	
Overweight	1 (100.0)	0 (0.0)	
Anoxia			0.273
Yes	98 (61.6)	61 (38.4)	
No	40 (54.1)	34 (45.9)	
**Cardiopulmonary bypass**			0.761
Yes	97 (59.9)	65 (40.1)	
No	41 (57.7)	30 (42.3)	
**Down syndrome**			< 0.001
Yes	39 (83.0)	8 (17.0)	
No	99 (53.2)	87 (46.8)	
**RACHS-1**			< 0.001
1	22 (37.9)	36 (62.1)	
2	60 (57.7)	44 (42.3)	
3 or 4	56 (78.9)	15 (21.1)	
**PH**			0.016
Yes	46 (71.9)	18 (28.1)	
No	92 (54.4)	77 (45.6)	
**Nitric oxide use**			0.081
Yes	10 (83.3)	2 (16.7)	
No	128 (57.9)	93 (42.1)	
**Continuous sedation**			< 0.001
Yes	102 (87.2)	15 (12.8)	
No	36 (31.0)	80 (69.0)	
**Age (months)**			< 0.001
< 12	60 (75.9)	19 (24.1)	
12 to 24	35 (66.0)	18 (34.0)	
> 24	43 (42.6)	58 (57.4)	

* Pearson's chi-square test

BMI=body mass index; CHD=congenital heart disease; MV=mechanical ventilation; PH=pulmonary hypertension; RACHS-1=Risk Adjustment for Congenital Heart Surgery

Patients with DS or under continuous sedation in the immediate postoperative period had more risk of prolonged MV (*P*<0.001, Table 3). Regarding extubation failure, children with the highest risk were younger than 12 months (*P*=0.048) or under prolonged MV (*P*=0.006) (Table 4; Table 5).

**Table 3.** Multivariate statistical analysis of independent factors associated with prolonged mechanical ventilation (n = 233).

Variable	RR (95% CI)	P-value*	RR (95% CI)	P-value*
**Neonate**		0.883		
Yes	1.03 (0.72 to 1.46)			
No	1.00			
**Down syndrome**		0.040		< 0.001
Yes	1.22 (1.01 to 1.47)		1.34 (1.14 to 1.57)	
No	1.00		1.00	
**RACHS-1**		0.152		
1	1.00			
2	1.18 (0.87 to 1.59)			
3 or 4	1.34 (0.98 to 1.84)			
**PH**		0.683		
Yes	1.04 (0.86 to 1.27)			
No	1.00			
**Nitric oxide use**		0.521		
Yes	0.92 (0.73 to 1.17)			
No	1.00			
**Continuous sedation**		< 0.001		< 0.001
Yes	2.43 (1.81 to 3.24)		2.71 (2.05 to 3.59)	
No	1.00		1.00	
**Age (months)**		0.301		
< 12	1.20 (0.95 to 1.51)			
12 to 24	1.15 (0.89 to 1.49)			
> 24	1.00			

* Pearson's chi-square test

CI=confidence interval; PH=pulmonary hypertension; RACHS-1=Risk Adjustment for Congenital Heart Surgery; RR=relative risk

**Table 4.** Bivariate analysis of clinical and demographic variables associated with extubation failure.

	Extubation failure		P-value
	Yes	No	
	N (%)	N (%)	
**Prolonged MV**			0.070*
Yes	17 (12.3)	121 (87.7)	
No	5 (5.3)	90 (94.7)	
**Neonate**			0.553**
Yes	1 (12.5)	7 (87.5)	
No	21 (9.3)	204 (90.7)	
**CHD diagnosis**			0.854**
Acyanotic	13 (9.0)	131 (91.0)	
Cyanotic	7 (12.3)	50 (87.7)	
Left obstructive	1 (5.0)	19 (95.0)	
Other	1 (8.3)	11 (91.7)	
**BMI category (according to age)**			0.538†
Low	21 (9.8)	194 (90.2)	
Appropriate	1 (5.9)	16 (94.1)	
Overweight	0 (0.0)	1 (100.0)	
**Anoxia**			0.333*
Yes	13 (8.2)	146 (91.8)	
No	9 (12.2)	65 (87.8)	
**Cardiopulmonary bypass**			0.732*
Yes	16 (9.9)	146 (90.1)	
No	6 (8.5)	65 (91.5)	
**Down syndrome**			0.047*
Yes	8 (17.0)	39 (83.0)	
No	14 (7.5)	172 (92.5)	
**RACHS-1**			0.081^†^
1	2 (3.4)	56 (96.6)	
2	11 (10.6)	93 (89.4)	
3	9 (13.2)	59 (86.8)	
4	0 (0.0)	3 (100.0)	
**Pulmonary hypertension**			0.047*
Yes	10 (15.6)	54 (84.4)	
No	12 (7.1)	157 (92.9)	
**Nitric oxide use**			0.018**
Yes	4 (33.3)	8 (66.7)	
No	18 (8.1)	203 (91.9)	
**Continuous sedation**			0.008*
Yes	17 (14.5)	100 (85.5)	
No	5 (4.3)	111 (95.7)	
**Age (months)**			< 0.001**
< 12	15 (19.0)	64 (81.0)	
12 to 24	5 (9.4)	48 (90.6)	
> 24	2 (2.0)	99 (98.0)	

*Pearson's chi-square test, **Fisher's exact test, ^†^Royston test for trend proportions

BMI=body mass index; CHD=congenital heart disease; MV=mechanical ventilation; RACHS-1=Risk Adjustment for Congenital Heart Surgery

**Table 5.** Multivariate analysis to identify factors independently associated with extubation failure.

	Initial multivariable model		Final multivariable model	
**Variable**	**RR (95% CI)**	***P*-value** *	**RR (95% CI)**	***P*-value** *
**Prolonged MV**		0.516		0.006
Yes	0.73 (0.28 to 1.89)		3.24 (1.40 to 7.54)	
No	1.00		1.00	
**Down syndrome**		0.203		
Yes	1.74 (0.74 to 4.10)			
No	1.00			
**RACHS-1**		0.550		
1	1.00			
2	1.80 (0.40 to 8.07)			
3 or 4	1.24 (0.22 to 6.82)			
**PH**		0.633		
Yes	1.25 (0.50 to 3.14)			
No	1.00			
**Continuous sedation**		0.139		
Yes	2.10 (0.78 to 5.63)			
No	1.00			
**Age (months)**		0.051		0.048
< 12	5.72 (1.34 to 24.48)		6.83 (1.42 to 32.80)	
12 to 24	3.27 (0.69 to 15.57)		4.06 (0.78 to 21.28)	
> 24	1.00		1.00	

* Pearson's chi-square test

CI=confidence interval; MV=mechanical ventilation; PH=pulmonary hypertension; RACHS-1=Risk Adjustment for Congenital Heart Surgery; RR=relative risk

## DISCUSSION

Patients with DS or continuous sedation in the immediate postoperative period had the highest risk of prolonged MV, whereas patients aged below 12 months or under prolonged MV had the highest risk of extubation failure. Moreover, our extubation failure rate was similar to previous studies (9.7%)^[5,13,14,15]^. A study from the United States of America observed that identifying patients with more risk of extubation failure and adopting more conservative extubation criteria could decrease this risk^[11]^.

Regarding the extubation site, most patients were extubated (fast-tracking) in the recovery unit; only a few were extubated in the OR. Joshi et al.^[12]^ suggested the fast-tracking in the OR, highlighting that patients with CPB for > 120 minutes, weighing < 5 kg, younger than 12 months, and with some associated syndrome would not be suitable for early extubation.

Polito et al.^[16]^ found that neonates, children needing surgical reoperation, care-related infection, and pulmonary complications were independent risk factors for prolonged MV. Another study identified that RACHS-1 category > 3, CPB for > 80 minutes, or anoxia for > 60 minutes were independent risk factors for prolonged MV^[17]^. In the present study, these factors were not significant for this variable.

We found that patients with DS or continuous sedation in the immediate postoperative period had the highest risk for prolonged MV, corroborating in part with Amula et al.^[18]^ results. The authors indicated that fewer sedative agents caused less respiratory system depression, reducing MV duration. They also suggested that cautious perioperative sedation may facilitate early extubation^[18]^. Joshi et al.^[12]^ showed different results: of the 18 patients with DS, 16 were extubated in the OR, and two within 48 hours; none required reintubation.

In the present study, patients with prolonged MV had more risk of extubation failure, corroborating Rooney et al.^[19]^ results. In another study, MV duration varied between centers: those where patients remained under MV for a short duration presented a low extubation failure rate^[20]^. When early extubation in the OR cannot be performed, MV maintains adequate gas exchange until the extubation, minimizing the risks of failure and reintubation. In contrast, the delayed extubation may increase infection risk, injury in the upper airway and lung parenchyma, sedation dependence, and mortality^[19,21]^. Furthermore, prolonged MV may impact hemodynamics (*i.e.*, heart rate, preload and afterload, and inotropism) in children^[22]^.

Regarding the spontaneous breathing trial, Ferreira et al.^[21]^ performed it in pressure support ventilation. They found that patients who performed the spontaneous breathing trial reduced the extubation failure rate compared with the control group. In the present study, the trial was conducted in the same mode, but the results were not significant.

Blackwood et al.^[23]^ stated that patients with risk factors for prolonged MV need specific sedation, analgesia, and weaning protocols to reduce MV duration and extubation failure. Wintch et al.^[24]^ found that 45% of patients with extubation failure were associated with agitation or delirium from the sedation. Therefore, reviewing analgesia and sedation protocols and following established guidelines could determine successful extubation^[18,23,24]^.

### Limitations

This study had some limitations. The medical records may have presented missed or incomplete data, limiting the analysis. We could not evaluate specific mortality using other scores (*i.e.*, Aristotle and the Society of Thoracic Surgeons-European Association for Cardio-Thoracic Surgery scores). Last, our study was conducted in a single center, despite being a reference in cardiology in the Northeast of Brazil.

## CONCLUSION

In the postoperative period of cardiac surgery, children and adolescents requiring prolonged MV used continuous sedation or had DS. A high extubation failure rate was evidenced in patients younger than 12 months or under prolonged MV. Thus, well-established protocols for sedation and extubation, especially in children younger than 12 months, may be the best strategy to prevent extubation failure and prolonged MV.

## Abbreviations, Acronyms & Symbols

BMI: Body mass index
CHD: Congenital heart disease
CI: Confidence interval
CPB: Cardiopulmonary bypass
DS: Down syndrome
ICU: Intensive care unit
MV: Mechanical ventilation
OR: Operating room
PH: Pulmonary hypertension
PROCAPE: Pronto-Socorro Cardiológico Universitário de Pernambuco Professor Luiz Tavares
RACHS-1: Risk Adjustment for Congenital Heart Surgery
RR: Relative risk
URCTPED: Unidade de Recuperação Cardio-Torácica Pediátrica
